# Cohort Profile of the NEIGE Study in Tokamachi City, Japan

**DOI:** 10.2188/jea.JE20190036

**Published:** 2020-07-05

**Authors:** Yugo Shobugawa, Hiroshi Murayama, Takeo Fujiwara, Shigeru Inoue

**Affiliations:** 1 Division of International Health, Niigata University Graduate School of Medical and Dental Sciences, Niigata, Japan; 2 Institute of Gerontology, The University of Tokyo, Tokyo, Japan; 3 Department of Global Health Promotion, Tokyo Medical and Dental University, Tokyo, Japan; 4 Department of Preventive Medicine and Public Health, Tokyo Medical University, Tokyo, Japan

**Keywords:** older adults, rural health, social determinants of health, cohort profile

## Abstract

**Background:**

Studies have shown that rural residents face disadvantages concerning medical access and socio-economic conditions. However, the social determinants of health among older people in rural areas are still unclear. The Neuron to Environmental Impact across Generations (NEIGE) study investigated the social determinants of health among older rural adults.

**Methods:**

A survey was conducted among the older residents of Tokamachi City, Japan. We randomly selected study participants (*N* = 1,346) from four stratified groups by age (65–74 and 75–84 years) and residential area (Tokamachi center [downtown] and Matsunoyama [mountain]). The survey collected data on socio-economic status, lifestyle, health, and neighborhood environment. Blood and urine sampling were also conducted, and physical activity was assessed. Magnetic resonance brain imaging (MRI) and Apo-E gene were also examined in the analysis.

**Results:**

In total, 527 people participated in the NEIGE study (participation rate: 39.2%). The average age of the participants was 73.5 (standard deviation, 5.6) years, and 47.3% were male. No differences in demographics were found between downtown and mountain residents, except for educational attainment, which was lower among mountain residents. Lifestyles were similar, except for the higher percentage of everyday drinkers (33.3–35.3%) in the mountain area. Concerning physical health, muscle mass, grip strength, and measured physical activity were significantly higher among mountain residents. However, gait speed and balance were better among downtown residents.

**Conclusion:**

The findings of the NEIGE study will help elucidate the social determinants of health in older rural adults in Japan, and emphasize the different outcomes between downtown and mountain areas.

## INTRODUCTION

Japan represents one of the most rapidly aging societies in the world, with this phenomenon more remarkable in rural areas.^1^ Older adults living in rural areas have limited medical resources, so they face disadvantages related to health.^2^^,^^3^ Lower health literacy and lower socio-economic status among older rural residents might also cause poor health outcomes.^4^^,^^5^

Universal health coverage is one of the most important issues promoted by the World Health Organization (WHO) and a key aspect of the United Nations’ Sustainable Development Goals (SDGs).^6^ Thus, the promotion of health among older adults residing in rural areas is essential.

The social determinants of health have been well investigated. However, some factors affect the health of older adults in urban and rural areas in different ways. For instance, social capital generally affect physical and psychological health positively.^7^^,^^8^ However, some studies have shown a null or opposite association in rural areas; for example, one study showed that particularized trust did not influence health in rural areas,^9^ while another one showed that bridging social capital was positively associated with depressive mood among women in rural settings.^9^^,^^10^ Since geographic, cultural, and historical contexts differ between urban and rural areas, the social determinants of health should be investigated and compared between rural and urban conditions. Furthermore, agricultural work has been determined to be an important social determinant of health for older rural adults. Several studies showed that gardening, which is included in agriculture, promotes mental health.^11^^–^^14^ However, there few studies have investigated the association between agricultural work and health for older adults.

Therefore, we launched a cohort study titled the Neuron to Environmental Impact across Generation (NEIGE) study in 2017 to investigate the social determinants of health among older rural residents. The present report partially describes the results of the NEIGE study’s baseline data.

## METHODS

### Study design, setting, and participants

The study population comprised individuals aged 65 through 84 years living in Tokamachi City, Niigata Prefecture, Japan. Tokamachi City is an agricultural city located 150 kilometers north of Tokyo. As of July 2017, the population was 54,515 (26,560 males and 27,955 females), with 20,089 people aged 65 years or older (proportion, 36.9%). The area of the city is approximately 590 square kilometers, with a low population density (87.8 persons per square kilometer). From the target age (65–84 years old) population (15,792 people) in the whole city, residents of the downtown and mountain areas of the city were examined, and their characteristics were compared to analyze their differences between social determinants of older health. The center of the city located downtown is the most populated area; conversely, the mountain area, set apart from the center, was selected as a representative of the least populated area. The distance between these two areas is 24 km. Tokamachi City receives heavy snowfall, with 9,978 cm (downtown) and 19,710 cm (mountain) in the winter of 2017. The name of the study, “NEIGE,” is also the French word for “snow.”

The target population in the selected downtown and mountain areas consisted of the 1,524 residents of Tokamachi, aged 65 to 84 years as of July 1, 2017. People with long-term care certification and those residing in nursing homes were excluded from the analysis. From the target population, 1,346 older adults were sampled. Then, invitations to participate in the study were sent to the sampled population via mail. We recruited participants using the stratified random sampling method based on the resident register. Equal numbers of participants were recruited for each age group (ie, 65–69, 70–74, 75–79, and 80–84) and also stratified by sex. As there is a large difference in environment and lifestyle between downtown residents and mountain residents, we also stratified the participants by residential area (downtown vs mountain). The sample size was calculated using the following formula:

$$n=p(1-p){\left(\frac{z}{E}\right)}^{2}$$

*n* = sample size required in each group
*z* = 1.96 for 95% CI
*E* = desired margin of error = 0.05
*p* = proportion of functional declining defined as long-term care insurance certification. (15% [roughly estimated] of the LTCI certification percentage gathered from the national data)
  $$n=0.15(1-0.15){\left(\frac{1.96}{0.05}\right)}^{2}$$
  n=196

Therefore, the minimum required sample size was 196 in each stratified group.

**Figure 1.**
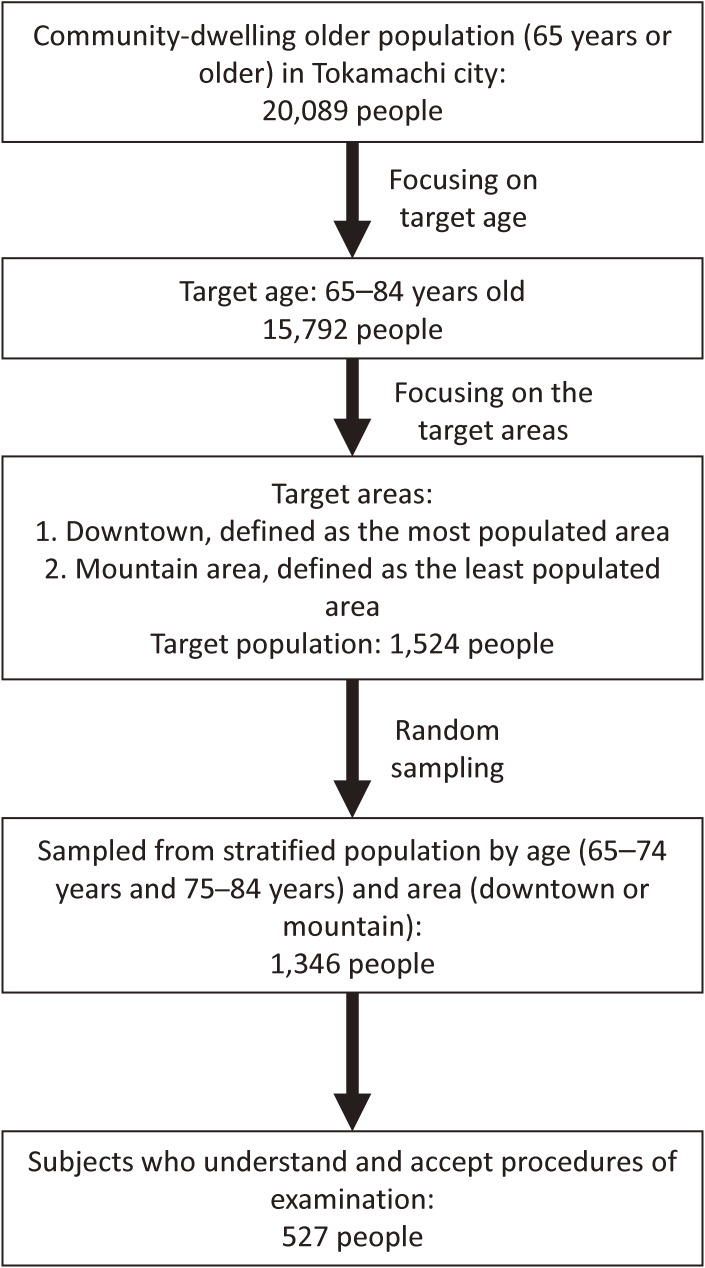
Flow diagram of the study participants.

A total of 527 people participated in the NEIGE study (Figure 1). There were no monetary or other incentives for participation. Before the survey, we informed participants of the research purpose and method.

### Baseline survey

The baseline survey was conducted in the mountain area in September and in the downtown area in October 2017. Participants completed the survey in person. The main items surveyed are summarized in Table 1.

**Table 1.** Summary of baseline survey items in the 2017 NEIGE study

Items	Description
**Demographics**	Age, sex, family members, marital status, equivalized income, educational attainment, employment status
**Lifestyle**	Agricultural work, smoking, alcohol consumption, sleep duration, physical activity measured by accelerometer, geographical range of daily activity measured by GPS
**Nutritional function**	Frequency of meat/fish consumption, frequency of vegetable/fruit consumption, food intake variety, biomarkers of nutrition, urinary sodium chloride
**Medical characteristics**	History of physician-diagnosed diseases (past or/and current), number of prescribed medications
**Body composition**	Body mass index, abdominal circumference, body composition, fat mass, muscle mass
**Cardiovascular function**	Blood pressure, heart rate
**Physical function**	Grip strength
	Gait speed
	Balance
**Psychological function**	Geriatric depression scale
**Cognitive function**	MMSE-J, analysis of para-hippocampal atrophy using MRI
**Activities of daily living**	Basic activities of daily living
	Higher-order competence of independence (TMIG-IC)
**Neighborhood environment**	Physical environment, social capital

*Demographics*: Age, sex, living arrangement, and socio-economic status. Socio-economic status includes educational attainment, income, and current employment.

*Lifestyle*: Agricultural work, smoking habit, alcohol consumption, sleep duration, and physical activity were measured using an accelerometer HJA-750C Active style Pro (Omron Healthcare, Kyoto, Japan). The geographical range of daily activities was captured using the Global Positioning System (GPS). To measure physical activity, a cutoff based on metabolic equivalents (METs) was used; ≤1.5 METs for sedentary behavior and ≥3.0 METs for moderate-to-vigorous physical activity. Accelerometer and GPS were prepared for auto recording; no handlings were needed for the participants.

*Nutritional function*: Dietary habits, including food intake variety, were assessed using the questionnaire (10 kinds of foods [eg, meats, vegetables, and milk]^15^^,^^16^). Biomarkers of nutrition were tested through blood samples, including evaluation of serum albumin, total cholesterol, and blood hemoglobin. Urinary sodium chloride was measured through laboratory testing to estimate salt intake per day.

*Medical characteristics*: History of physician-diagnosed diseases (past or/and current) and number of prescribed medications were obtained by interviewers (medical doctors or nurses).

*Body composition*: Body mass index (BMI), abdominal circumference, fat mass, and muscle mass. BMI was calculated from measured body height and body weight. Fat mass and muscle mass were estimated from bioelectrical impedance analysis using a body composition analyzer MC-780A (TANITA Corporation, Tokyo, Japan). All measurement was conducted by the research staff (no self-measurement).

*Cardiovascular function*: Blood pressure and heart rate were measured using a blood pressure monitor UA-1020 (A & D Medical, Saitama, Japan). The measurement was conducted by health staff (medical doctor or nurse).

*Physical function*: Grip strength, 5-meter usual gait speed, and standing time on one foot with eyes open (maximum: 60 seconds). Grip strength was measured twice in each hand using a dynamometer. Gait speed was calculated as time (seconds) taken for a 5-meter walk. Balance was evaluated based on standing time on one foot. The measurement was conducted by trained staff.

*Psychological function*: The Geriatric Depression Scale (GDS) short-form (15 items)^17^^–^^20^ and self-rated health.

*Cognitive function*: The Mini-Mental State Examination Japanese version (MMSE-J)^21^ and analysis of para-hippocampal atrophy using Magnetic resonance brain image.^22^ VSRAD (Voxel-based Specific Regional analysis system for Alzheimer’s Disease) was applied to study para-hippocampal atrophy.^23^ MMSE-J was evaluated by trained staff.

*Activities of daily living (ADL)*: Basic ADL and higher-level functional capacity. The Tokyo Metropolitan Institute of Gerontology Index of Competence, which consists of three subscales (instrumental self-maintenance, intellectual activity, and social role) were used.^24^

*Neighborhood environment*: neighborhood living environment and neighborhood social capital, including civic participation, general trust, and attachment to neighbors. To measure social capital, a method from the previous study was partially applied.^25^

We also investigated genetic factors including polymorphism of the Apo-E gene and sense of hearing and smell. Remaining blood samples were kept for further analyses.

### Follow-up outcome surveys

We will follow-up on three survey items: health outcomes, lifespan, and long-term care insurance (LTCI) certification. To measure individual changes in health outcomes, we will conduct a follow-up survey every 3 years in the same manner as the baseline survey. To follow survival time and LTCI certification since the baseline survey, we will confirm information from Tokamachi City annually.

### Ethical considerations

The study protocol was reviewed and approved by the Ethics Committee of Niigata University on November 25, 2016 (approval number: 2666). Written informed consent was obtained from all study participants.

### Statistical analysis

To describe the baseline characteristics of the study participants, we compared the main measures among the participants according to age (65–74 vs 75–84 years old) and residential area (downtown vs mountain) using the chi-square test for nominal variables, the Kruskal-Wallis test for ordinal variables, and *t*-test for continuous variables. Statistical significance was set as a *P* value of less than 0.05 on a two-tailed test. All the analyses were performed using STATA 15 (Stata Corp, College Station, TX, USA).

## RESULTS

Table 2 displays results concerning the demographic, life style, physical and mental health status. The agricultural working rate was significantly higher among mountain residents in both age groups (both *P* < 0.001). The marriage rate was lower among downtown residents aged 65–74 (*P* = 0.03) than among mountain residents (Table 3), while educational attainment was higher among downtown residents than mountain residents in both age groups. Alcohol consumption was significantly higher in the mountain area in the 75–84 group (*P* = 0.012). Objectively measured physical activity showed that sedentary time was longer among downtown residents (452.0 [standard deviation {SD}, 122.2] vs 412.0 [SD, 140.4], *P* = 0.009), and moderate-to-vigorous physical activity time was shorter (57.2 [SD, 36.3] vs 71.8 [SD, 45.9], *P* = 0.002) among downtown residents aged 65–74. For body composition, muscle mass was significantly higher among the mountain residents and fat mass was higher among downtown residents. Blood pressure did not differ between downtown and mountain residents. Urinary sodium chloride, which can be used to estimate salt intake, was significantly higher among downtown residents in both age groups. Grip strength was stronger among mountain residents, while gait speed was faster among downtown residents of both age groups. Finally, balance was better among downtown residents in both age groups. The results of the MRI are not shown because validation of the obtained data is ongoing.

**Table 2.** Summary of the main items surveyed according to sex and age group in the NEIGE study

		Total	Male		Female	
			65–74 years	75–84 years	65–74 years	75–84 years
		*N* = 527	*N* = 149	*N* = 100	*N* = 102	*N* = 104
**Demographics**						
Age	Mean (SD)	73.5 (5.6)	69.5 (2.7)	79.5 (2.9)	69.3 (2.8)	79.1 (2.7)
Sex	Men, %	47.3	—	—	—	—
Family members	Living alone, %	8.9	4.0	4.0	9.4	18.5
Marital status	Married, %	80.5	94.0	91.0	79.3	56.3
Equivalized annual income, JPY	2.00 million or above	54.2	66.5	46.0	56.6	42.9
Educational attainment, years	10 years or longer, %	61.7	83.9	56.0	62.9	36.9
Employment status	Currently employed, %	41.2	53.0	38.0	46.5	21.9

**Lifestyle**						
Agricultural work	Currently working, %	58.4	67.1	56.0	52.8	57.1
Smoking	Current smoker, %	8.9	18.1	13.0	3.8	0.8
Alcohol consumption	Every day or sometimes, %	53.2	81.9	73.0	38.4	20.1
Sleeping duration	Mean (SD)	7.7 (1.2)	7.7 (1.1)	8.5 (1.2)	7.1 (1.0)	7.6 (1.1)
Physical activity	Sedentary time, min/day	445.4 (129.7)	456.8 (141.9)	486.1 (136.7)	408.8 (120.1)	447.5 (107.0)
	Moderate-to-vigorous physical activity time, min/day	52.3 (39.9)	65.9 (43.4)	38.6 (33.4)	63.3 (40.7)	31.4 (24.2)

**Nutritional function**						
Frequency of meat/fish consumption	Everyday, %	58.4	54.4	57.0	54.1	70.6
Frequency of vegetable/fruit consumption	Everyday, %	89.0	91.3	86.0	86.2	92.4
Food intake variety	Mean (SD)	5.5 (2.2)	5.2 (2.2)	5.3 (2.5)	5.5 (2.2)	6.1 (1.9)
Biomarkers of nutrition	Serum albumin, g/dL Mean (SD)	4.4 (0.3)	4.4 (0.3)	4.3 (0.3)	4.5 (0.3)	4.4 (0.3)
	Total cholesterol, mg/dL Mean (SD)	203 (33)	199 (33)	190 (34)	214 (30)	207 (33)
	Blood hemoglobin, g/dL Mean (SD)	13.6 (1.4)	14.6 (1.2)	14.0 (1.3)	13.2 (1.0)	12.7 (1.2)
Urinary sodium chloride, g per day	Mean (SD)	9.7 (2.1)	9.9 (2.1)	9.9 (2.3)	9.7 (2.0)	9.5 (2.1)

**Body composition**						
Body mass index, kg/cm^2^	Mean (SD)	22.1 (3.0)	22.6 (2.5)	22.8 (2.6)	21.7 (3.2)	21.5 (3.3)
Abdominal circumference, cm	Mean (SD)	83.0 (9.2)	84.9 (7.5)	86.9 (8.1)	80.7 (9.8)	80.5 (9.5)
Fat mass, %	Mean (SD)	24.1 (8.3)	18.1 (5.6)	21.2 (6.2)	28.3 (7.7)	28.3 (7.9)
Muscle mass, kg	Mean (SD)	39.5 (7.7)	47.8 (4.3)	44.5 (5.1)	34.0 (2.7)	32.2 (2.9)

**Cardiovascular function**						
Systolic blood pressure, mm Hg	Mean (SD)	138 (19)	138 (17)	141 (18)	135 (20)	140 (20)
Diastolic blood pressure, mm Hg	Mean (SD)	82 (11)	85 (11)	81 (11)	82 (10)	81 (11)
Heart rate, beats/min	Mean (SD)	70 (12)	69 (11)	69 (11)	70 (10)	73 (13)

**Physical function**						
Grip strength, kg	Mean (SD)	30.1 (8.2)	38.7 (5.9)	33.6 (6.5)	25.3 (3.7)	22.6 (3.7)
Gait speed, m/s	Mean (SD)	1.3 (0.3)	1.3 (0.2)	1.2 (0.3)	1.4 (0.2)	1.2 (0.3)
Standing time on 1 foot with eyes open, seconds	Mean (SD)	34.5 (23.3)	42.7 (21.8)	25.7 (22.3)	41.7 (21.4)	25.1 (21.9)

**Psychological function**						
Geriatric Depression Scale	Mean (SD)	2.8 (2.7)	2.4 (2.3)	2.5 (2.3)	2.9 (2.9)	3.4 (3.1)

**Cognitive function**						
Mini-Mental Status Examination Japanese version	Mean (SD)	27.0 (2.6)	26.6 (2.9)	27.2 (2.6)	26.7 (2.6)	27.6 (2.2)

**Activities of daily living (ADL)**						
Instrumental self-maintenance (range: 0–5)	Full points, %	53.3	29.5	27.0	81.8	67.2
Intellectual activity (range: 0–4)	Full points, %	68.1	76.5	69.0	63.5	63
Social role (range: 0–4)	Full points, %	61.3	53.0	50.0	71.7	67.2

**Neighborhood environment**						
Physical environment:						
park or walking road	Yes, %	63.2	58.4	70.0	61.6	65.6
Grocery	Yes, %	66.7	59.7	75.0	69.2	65.3
Social capital						
Civic participation	None, %	25.2	17.5	38.0	20.8	30.3
	One, %	29.4	36.2	24.0	33.3	20.2
	Two or more, %	45.4	46.3	38.0	45.9	49.6
General trust	Yes, %	80.1	84.6	83.0	73.6	80.7
Attachment to neighbor	Yes, %	88.4	91.3	91.0	83.0	89.9

**Table 3.** Summary of the main measures surveyed divided by residential area and age group in the NEIGE study

		Total	Urban (downtown)		Rural (mountain)		*P* between downtown and mountain	
			65–74 years	75–84 years	65–74 years	75–84 years	Among 65–74 years	Among 75–84 years
		*N* = 527	*N* = 155	*N* = 126	*N* = 153	*N* = 93		
**Demographics**								
Age	Mean (SD)	73.5 (5.6)	69.5 (2.8)	79.2 (2.8)	69.2 (2.7)	79.3 (2.8)	ns	ns
Sex	Male, %	47.3	41.9	43.7	54.9	48.4	0.023	ns
Family members	Living alone, %	8.9	6.5	9.5	7.2	15	ns	ns
Marital status	Married, %	80.5	81.3	70.6	91.5	74.2	0.03	ns
Equivalized income, JPY	2.00 million or above	54.2	63.3	52.4	59.5	33.4	ns	ns
Educational attainment, years	10 years or longer, %	61.7	81.9	54.8	64.1	33.4	0.002	0.007
Employment status	Currently employed, %	41.2	54.2	27.8	45.1	31.2	ns	0.045

**Lifestyle**								
Agricultural work	Currently working, %	58.4	27.1	34.1	92.8	87.1	<0.001	<0.001
Smoking	Current smoker, %	8.9	9.7	6.4	11.8	6.5	ns	ns
Alcohol consumption	Every day or sometimes, %	53.2	62.0	42.9	56.9	46.2	ns	0.012
Sleep duration	Hours, Mean (SD)	7.7 (1.2)	7.4 (1.1)	7.9 (1.2)	7.4 (1.1)	8.2 (1.2)	ns	ns
Physical activity	Sedentary time, min/day Mean (SD)	445.4 (129.7)	452.0 (122.2)	477.8 (127.2)	412.0 (140.4)	448.5 (114.9)	0.009	ns
	Moderate-to-vigorous physical activity time, min/day Mean (SD)	52.3 (39.9)	57.2 (36.3)	34.2 (30.0)	71.8 (45.9)	35.2 (27.4)	0.002	ns

**Nutritional function**								
Frequency of meat/fish consumption	Everyday, %	58.4	58.1	65.1	50.3	63.4	ns	ns
Frequency of vegetable/fruit consumption	Everyday, %	89.0	86.5	90.5	90.9	88.2	ns	ns
Food intake variety	Mean (SD)	5.5 (2.2)	5.3 (2.3)	5.8 (2.2)	5.5 (2.1)	5.7 (2.3)	ns	ns
Biomarkers of nutrition	Serum albumin, g/dL Mean (SD)	4.4 (0.3)	4.4 (0.3)	4.4 (0.3)	4.5 (0.3)	4.4 (0.3)	ns	ns
	Total cholesterol, mg/dL Mean (SD)	203 (33)	210.6 (33.7)	200.9 (37.1)	201.9 (30.4)	197.0 (30.4)	0.018	ns
	Blood hemoglobin, g/dL Mean (SD)	13.6 (1.4)	13.8 (1.3)	13.2 (1.4)	13.2 (1.4)	13.4 (1.3)	ns	ns
Urinary sodium chloride, g per day	Mean (SD)	9.7 (2.1)	10.1 (2.0)	9.9 (2.0)	9.4 (2.0)	9.3 (2.3)	0.003	0.038

**Body composition**								
Body mass index, kg/cm^2^	Mean (SD)	22.1 (3.0)	22.3 (2.9)	22.3 (2.9)	22.0 (3.0)	21.9 (3.3)	ns	ns
Abdominal circumference, cm	Mean (SD)	83.0 (9.2)	82.0 (9.4)	83.2 (9.6)	83.5 (8.5)	83.7 (9.4)	ns	ns
Fat mass, %	Mean (SD)	24.1 (8.3)	25.1 (8.5)	26.0 (7.1)	21.6 (8.2)	23.7 (9.0)	<0.001	0.04
Muscle mass, kg	Mean (SD)	39.5 (7.7)	70.8 (8.1)	69.5 (9.2)	74.2 (7.8)	71.5 (11.3)	<0.001	ns

**Cardiovascular function**								
Systolic blood pressure, mm Hg	Mean (SD)	138 (19)	136.1 (16.6)	139.1 (18.7)	137.1 (20.8)	142.1 (19.9)	ns	ns
Diastolic blood pressure, mm Hg	Mean (SD)	82 (11)	83.7 (10.1)	79.8 (10.6)	83.2 (11.2)	82.1 (10.9)	ns	ns
Heart rate, beats/min	Mean (SD)	70 (12)	70.1 (11.5)	71.4 (11.5)	68.7 (10.1)	70.7 (13.6)	ns	ns

**Physical function**								
Grip strength, kg	Mean (SD)	30.1 (8.2)	30.2 (7.9)	27.0 (7.7)	33.5 (8.3)	28.4 (7.4)	0.001	ns
Gait speed, m/s	Mean (SD)	1.3 (0.3)	1.4 (0.2)	1.3 (0.3)	1.3 (0.2)	1.1 (0.2)	<0.001	<0.001
Balance, seconds	Mean (SD)	34.5 (23.3)	48.4 (18.8)	28.2 (22.2)	35.9 (22.5)	21.3 (21.3)	<0.001	0.025

**Psychological function**								
Geriatric Depression Scale	Mean (SD)	2.8 (2.7)	2.9 (2.9)	3.3 (3.0)	2.4 (2.3)	2.6 (2.4)	ns	ns

**Cognitive function**								
Mini-Mental Status Examination Japanese version	Mean (SD)	27.0 (2.6)	2.9 (2.9)	3.3 (3.0)	2.4 (2.3)	2.6 (2.4)	ns	ns

**Activities of daily living (ADL)**								
Instrumental self-maintenance (range: 0–5)	full points, %	53.3	56.8	47.6	56.2	50.5	ns	0.038
Intellectual activity (range: 0–4)	full points, %	68.1	76.8	68.3	62.8	62.4	0.06	ns
Social role (range: 0–4)	full points, %	61.3	60.7	59.5	64.7	59.1	ns	ns

**Neighborhood environment**								
Physical environment								
park or walking road	Yes, %	63.2	77.4	75.4	42.5	57.0	<0.001	0.004
Grocery	Yes, %	66.7	87.1	81.0	41.8	54.4	<0.001	<0.001
Social capital								
Civic participation	None, %	25.2	15.5	27.0	22.9	43.0		
	One, %	29.4	33.6	22.2	36.0	21.5		
	Two or more, %	45.4	51.0	50.8	41.2	35.5	ns	0.032
General trust	Yes, %	80.1	79.4	79.4	78.4	85.0	ns	ns
Attachment to neighbor	Yes, %	88.4	85.8	88.9	88.2	92.5	ns	ns

## DISCUSSION

The NEIGE study is a cohort study, and this report partially describes data at the baseline survey. Our findings showed that muscle mass and grip strength were higher among the mountain residents. Although these residents face disadvantages concerning access to medical care and socio-economic status (including educational attainment and household income), which can cause lower instrumental ADL, cognitive impairment, or even higher mortality rates,^26^ rural mountain life and agricultural work can often improve residents’ health. Our baseline data indicates that older mountain residents generally had better physical capacities than their downtown counterparts. However, the results of the gait speed and balance tests were poorer for mountain residents.

Compared to the participants of other cohort studies, such as the Kashiwa Cohort Study and NILS-LSA, hand grip strength was found to be stronger in older adults from Tokamachi.^27^^,^^28^ However, the gait speed of the participants in the other studies was similar to or faster than the participants from Tokamachi. The burden of agricultural work might decrease scores for several physical functions because of curved posture, lumbago, and knee pain. Percent fat mass was higher for participants, specifically older age participants (75 or older), in Tokamachi than for those in NILS-LSA.^28^ This difference might be caused because of the maintenance of muscle mass due to the continuance of agricultural work even after the standard retirement age (65 years old) in rural areas. However, this requires further investigation. In our study, lower GDS scores were presented among older adults in the mountain area, but were statistically not significant. This finding might support previous studies showing that gardening reduces stress and anxiety.^11^^–^^14^

The strengths of the NEIGE study include the following. First, the study is a comprehensive investigation of physical and mental functions, social factors, spatial factors, genetic factors, and objective measurements, such as accelerometer and magnetic resonance imaging. Such multiple factors can be utilized to elucidate significant causes of functional decline and frailty. Second, geographical records and objectively measured physical activity data were used. These data make it possible to evaluate physical activity in more detail using objective rather than subjective measurements. Third, face-to-face interviews were used to complete the baseline surveys; this is a more effective method for completing the dataset as compared to questionnaire surveys, which may have missing data. Fourth, we will follow-up on the participants through face-to-face interviews and collect data on lifespan and LTCI certification. Older rural residents rarely move to other areas; thus, we do not anticipate much difficulty following-up on the study participants. Follow-up data can elucidate longitudinally social determinants of health and mortality.

The limitations of the study are as follows. First, the participants were randomly selected within the target area, but the participants were volunteers who could come to the survey location. Thus, the people who participated in the survey were possibly healthier than average for this population. Second, the effect of agricultural work on mental health needs to be further investigated.

In conclusion, the NEIGE study was launched in 2017 to evaluate the social determinants of health among older Japanese residents from rural areas. An additional follow-up survey will be conducted 3 years after the baseline survey. Information on lifespan and LCTI certification will be also collected. The NEIGE study will contribute to designing health policies that promote healthy and active lifestyles among older adults in Japan.
